# Comparison of Informal Care Time and Costs in Different Age-Related Dementias: A Review

**DOI:** 10.1155/2013/852368

**Published:** 2012-12-05

**Authors:** Nadège Costa, Laura Ferlicoq, Hélène Derumeaux-Burel, Thomas Rapp, Valérie Garnault, Sophie Gillette-Guyonnet, Sandrine Andrieu, Bruno Vellas, Michel Lamure, Alain Grand, Laurent Molinier

**Affiliations:** ^1^UMR1027, Inserm, 31073 Toulouse, France; ^2^UMR1027, University of Toulouse III, 31073 Toulouse, France; ^3^Medical Information Department, University Hospital of Toulouse, 31059 Toulouse, France; ^4^Département d'Information Médicale, Hôtel-Dieu Saint-Jacques, 2 rue Viguerie, TSA 80035, 31059 Toulouse Cedex 9, France; ^5^LIRAES, University of Paris Descartes, 75005 Paris, France; ^6^Department of Geriatric Medicine, University Hospital of Toulouse, Gérontopôle of Toulouse, 31059 Toulouse, France; ^7^Department of Epidemiology and Public Health, University Hospital of Toulouse, 31059 Toulouse, France; ^8^EDISS, University of Lyon I, 69100 Villeurbanne, France

## Abstract

*Objectives*. Age-related dementia is a progressive degenerative brain syndrome whose prevalence increases with age. Dementias cause a substantial burden on society and on families who provide informal care. This study aims to review the relevant papers to compare informal care time and costs in different dementias. *Methods*. A bibliographic search was performed on an international medical literature database (MEDLINE). All studies which assessed the social economic burden of different dementias were selected. Informal care time and costs were analyzed in three care settings by disease stages. *Results*. 21 studies met our criteria. Mean informal care time was 55.73 h per week for Alzheimer disease and 15.8 h per week for Parkinson disease (*P* = 0.0076), and the associated mean annual informal costs were $17,492 versus $3,284, respectively (*P* = 0.0393). *Conclusion*. There is a lack of data about informal care time and costs among other dementias than AD or PD. Globally, AD is the most costly in terms of informal care costs than PD, $17,492 versus $3,284, respectively.

## 1. Introduction

Dementia is a collective name for progressive degenerative brain syndromes which affect memory, thinking, behavior, and emotion. Symptoms may include loss of memory, difficulty in finding the right word or understanding what people are saying, difficulty in performing previously routine tasks, personality, and mood changes [1]. Age-related dementia refers to a group of dementias whose prevalence increases with age. Alzheimer disease (AD), Parkinson disease (PD), vascular dementia (VD), Lewy body dementia (DLB), and frontotemporal dementia (FTD) are the most common age-related dementias.

Dementia is one of the major causes of disability and dependence among older people worldwide. It is overwhelming not only for the people who suffer from it but also for their caregivers and families. These diseases cause a substantial burden on society and especially on families who are often required to endorse the informal caregiver's role. Informal care can be defined as unpaid care provided to parents or friends who present functional or/and cognitive disability [2]. However, this definition can be discussed and particularly the notion of “unpaid.” According to van den Berg et al., informal caregivers may receive some form of payment. They consider being informal care “when the caregiver would not want to care for someone outside of his social environment for a similar wage” [3]. Thus, informal care can be defined as “nearly” unpaid care provided to someone inside the social environment of informal caregiver. The burden of informal care can be the point to an early placement in institution which leads to an increase of total costs [4, 5].

With the gross of the worldwide life expectancy, dementia will have a more important impact on society and family burden. Currently, the number of people living with dementia worldwide in 2011 is nearly 35.6 million, increasing to 115.4 million by 2050 [6]. The worldwide economic burden associated with these diseases is estimated at US$ 604 billion. Informal care costs take an important part in the economic burden of dementia. Indeed, Mauskopf and Mucha estimated at 36 to 85% the part of informal care in total costs of dementia [7]. Nevertheless, no studies have compared the economic burden of informal costs between different age-related dementias as AD, PD, VD, FTD, or DLB.

The aim of this study is to review relevant papers in order to compare informal care time and informal costs in AD, PD, DLB, FTD, and VD. Informal care time and costs will be analyzed according to severity stages. We focused on data collected at a given point.

## 2. Methods

### 2.1. Cost Study

#### 2.1.1. Age-Related Dementias

Age-related dementias concern patients aged over 60 years and can refer to AD, PD, VD DLB, and FTD.

AD is a degenerative brain syndrome characterized by a progressive decline in memory, thinking, comprehension, calculation, language, learning capacity, and judgment sufficient to impair personal activities of daily living. It is the 1st cause of general and degenerative dementia (60–70%). Diagnosis is made using NINCDS-ADRDA criteria [8].

PD is a progressive neurodegenerative disorder, which affects movement or the control of movement, including speech and “body language.” Diagnosis is made using UKPDRS criteria [9]. 

VD is a general term describing problems with reasoning, planning, judgment, memory, and other thought processes resulting from an impaired blood flow in brain. It is the 2nd cause of general dementia after AD: 10 to 20%. Diagnosis is made using NINCDS-AIREN criteria [10]. 

DLB is a progressive degenerative disease or syndrome of the brain. It shares—and sometimes overlaps—symptoms with several diseases, especially AD and PD. It is the 2nd cause of degenerative dementia after AD (15–25%). Diagnosis is made using McKeith et al. criteria [11].

FTD is marked by dramatic changes in personality, behavior (loss of inhibition, apathy, social withdrawal, hyperorality ritualistic compulsive behaviors) and some thought processes. FTD leads to immobility and loss of speech and expression. It represents 10% of general dementia cases [12].

#### 2.1.2. Disease Severity

As informal care time and costs increase with disease severity, this information must be provided [34–36]. Disease severity is usually measured with the Mini-Mental State Examination (MMSE) [37] for AD and VD and with the Hoehn and Yarh criteria (HY) for PD [38]. Severity of AD and VD is often classified into mild, moderate, and severe stages. However in PD, HY criteria are often classified with numbers ranging from 1 to 5. To be as homogeneous as possible in this paper, levels 1 and 2 of HY criteria were assimilated as mild, level 3 as moderate, level 4 as moderate to severe, and level 5 as severe.

#### 2.1.3. Place of Living

The place of living must be taken into consideration because different cost components may vary depending on whether the patient lives at home or in institution [39, 40]. Indeed, when patients live in institution, a large share of costs is attributable to direct medical costs while, for patients living at home, a large share of costs is attributable to informal costs (classified as direct nonmedical costs or indirect costs) [41, 42].

#### 2.1.4. Informal Care Time Measurement

Methods for estimating resource consumption vary depending on the available data.

Some methods are well described in the international literature. The diary method which is perceived as the gold standard for the measurement of time used entails respondents that are asked to write down all their activities during a specific period [43–45] because it collects time allocation data in structured way and involves a relatively short recall period. The recall method entails respondents being asked how much time they spent on a list of activities during, for example, the previous day or week.

Various instruments exist to measure informal caregiving time, such as the Caregiver Activity Time Survey (CATS) [46], the Caregiver Activity Survey (CAS) [47], and the Resource Utilization in Dementia (RUD) [48].

Finally, informal care time can be measured for different activities as activity of daily living (ADL), instrumental activity of daily living (IADL), and supervision [48].

In this paper, if informal care time was not available, we divided annual informal cost by unit cost of one hour of informal care. It gave us hours spent on informal care per year. We assume that informal caregivers provided care seven days a week and 48 weeks by year, because respite structures and other informal or formal caregivers can take care of the patients during four weeks by year. If only annual results were available, it is then divided by 48 to obtain weekly hours of informal care.

#### 2.1.5. Informal Care Costing

Informal care plays a substantial role in the total care for people with chronic disease. Several methods are used to value the shadow price of informal care time.

Opportunity cost approach consists to ask the caregiver to identify the opportunities forgone as a result of caregiving [7]. It attempts to place a monetary value on the alternative use of carer time. In assessing the financial opportunity cost, different values are assigned to caring depending on whether the alternative use of time spent caring is paid as employment or leisure time forgone. Valuation will depend on the age, education, and previous work experience of the carer. If caregiver is nonworking, informal care time is valued with leisure time, as a percentage of work time usually between 25% and 33% [49].

Replacement cost approach values the time spent on caregiving at the labour market price of a close substitute. Informal care time is valued at the wage rate or market price of a professional caregiver [50]. This approach allows the division of informal care into several tasks and thus to value informal care time with different average wages which can be based on the hourly rates for nurse's aides, cleaners, book-keepers, and social workers [51].

These two methods are the mainly used [52, 53], but other methods as the well-being valuation (WBV) [54], the contingent valuation method (CVM) [55], or the conjoint measurement (CM) [56] are available to value informal care time.

Van den Berg and Ferrer-I-Carbonell present an alternative valuation method in which the costs of providing informal care are valued in terms of loss of well-being suffered by the informal caregiver. This approach consists in a first step to estimate the effect of providing informal care and of income on individual's subjective well-being and in a second time to estimate the necessary income (compensating variation) to maintain the same level of informal caregiver's well-being after providing an additional hour of informal care. This compensating variation is taken as the monetary value of informal care [54].

The CVM is capable of deriving the net value per hour of informal caregiving from the perspective of the informal caregiver [55]. CVM is based on the work of Hicks, who developed measures of losses and keeping utility constant [57]. This method is sensitive to the real individual's preferences [55].

The CM method (or conjoint analysis) is a method for the analysis of respondent's preferences over a set of multiattribute alternatives [56]. The situation differs according to some dimensions called attributes. If the price or cost is included as an attribute, it is possible to derive implicit prices or costs for each of the other dimensions. So a monetary value of the good in question can be derived. Conjoint measurement includes both the informal caregiver's opportunity costs of time and the derived (direct) utility and (direct) disutility of providing care [56].

Moreover, informal care costs can be assessed using its percentage in the total cost (TC) of the disease. Total costs included direct medical costs (e.g., institution costs, medications, inpatients, visits etc.), direct nonmedical costs (e.g., rehabilitation, home help, and transportation), and indirect costs (i.e., productivity losses). Usually, informal care costs are classified as direct nonmedical costs if informal caregiver is considered as a proxy of home help or they can be classified as indirect costs if opportunity costs approach is used. 

#### 2.1.6. Statistical Analysis

A comparison between dementia's informal care time and costs was realized. Box plots were performed to measure informal care time and cost's dispersions between dementias. Bivariate analyses were performed to compare informal care time and informal costs between dementias. Student's tests were used to compare samples when the sample distribution was normal and variances were equal. Otherwise, nonparametric tests as Wilcoxon test were used. These tests were performed with STATA 12 software.

### 2.2. Literature Review

#### 2.2.1. Study Selection

A bibliographic search was performed on an international medical literature database (MEDLINE). All studies which assessed social economic burden of AD, PD, VD, FTD, or DLB were selected. In order to be as exhaustive as possible, five combinations using keywords were employed: ((“Societal costs” OR “Informal costs” OR “Cost of illness”) AND “Alzheimer disease”); ((“Societal costs” OR “Informal costs” OR “Cost of illness”) AND “Parkinson disease”); ((“Societal costs” OR “Informal costs” OR “Cost of illness”) AND “Vascular dementia”); ((“Societal costs” OR “Informal costs” OR “Cost of illness”) AND “Fronto-temporal dementia”); ((“Societal costs” OR “Informal costs” OR “Cost of illness”) AND “Dementia with Lewy Body”). This search provided us 851 papers. We kept the 735 papers written in English language. Among them, we selected articles whose title contained “Alzheimer disease” or “Parkinson disease” or “Vascular dementia” or “Fronto-temporal dementia” or “Dementia with Lewy Body” or “Dementia” AND “ Societal Costs” or “Cost of illness” or “Costs” (447 papers were removed).

**Figure 1:**
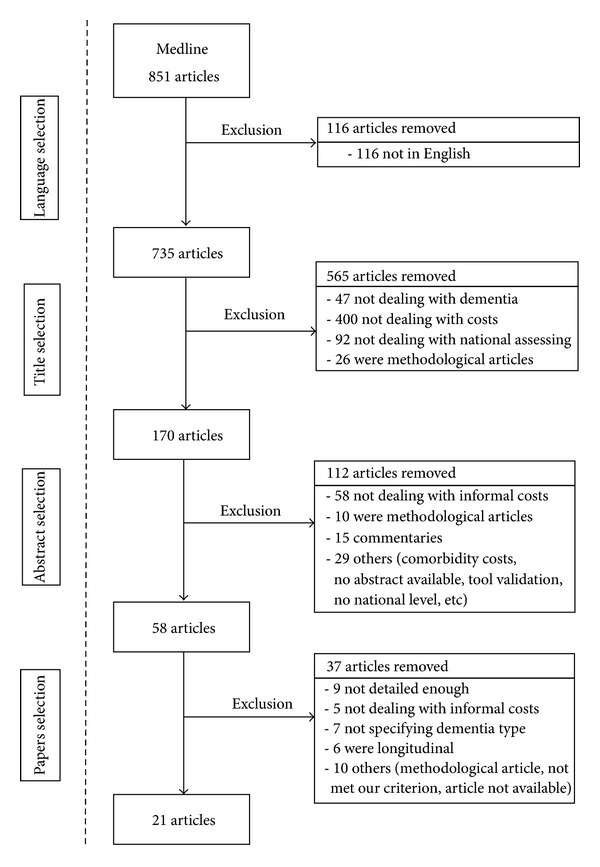
Literature search and selection process.

All studies assessing informal time and costs on different dementias at a national level were selected. Ninety-two papers were excluded because they did not assess results at a national level. One hundred and seventy abstracts were first selected, and 58 of them underwent a subsequent full paper reading. Among these, 6 papers were removed because they were longitudinal studies and thus analyzed costs and severity evolution and not cost and severity at a given time, so they do not correspond to our objective that was to estimate costs and severity at a given time [58]. Paper selection provided 21 articles.  Figure 1 illustrates the literature search and selection process and presents reasons for excluding studies.

#### 2.2.2. Study Review

A systematic review was performed. One author (N. Costa) selected abstracts. Six methodologists read the 58 papers retrieved by the search strategy and reviewed the 21 selected papers.

## 3. Results

Twenty-one studies met our criteria, and their characteristics are summarized in Table 1. Fifteen studies focused on AD, among them, 8 were carried out in Europe [13–20], 4 in North America [21–24], 2 in Asia [25, 26], and 1 in South America [27]. Five studies focused on PD, among them, 3 were carried out in Europe [28–30], 1 in North America [31], and 1 in Asia [32]. One study carried out in Europe focused on VD [33]. No studies were retrieved about informal costs and time of FTD and DLB. In 19 studies, sample size varied from 42 to 948 [13–20, 22–32] and two studies did not specify the number of patients included [21, 33]. Studies focused on PD, included patient, between 63.60 and 71.77 years [28–32], and studies focused on AD selected patients between 65.95 and 81.50 years [13–21, 24–27]. Study on VD did not specify the average of patients' age [33].

### 3.1. Measurement and Costing of Informal Care

Three studies used the diary method to gather time spent on informal care [21, 22, 26]. Among them, two studies recorded data through a calendar and caregivers were asked to report time spent by themselves and other caregivers on caregiving tasks and only regarding Alzheimer disease [21, 22]. This data were collected through 12 monthly interviews. Zencir et al. used daily time shit and asked caregivers to report the time spent on caregiving task on a 15-day period [26].

Sixteen studies used the recall method to gather activity data [13–20, 23–25, 27, 29–32]. Among them, 11 studies used ad hoc questionnaires or adapted questionnaires from another studies [13, 14, 18, 20, 23, 25, 27, 29–32], 3 studies used the RUD [16, 17, 19], and two studies used face to face or telephone interviews [15, 24].

Two studies have not defined the method used to gather time spent on caregiving tasks [28, 33].

The recall period was one week for one study [18], one month for 6 studies [13, 17, 22–25], 3 months for 5 studies [14, 15, 27, 30, 32], 6 months for 2 studies [16, 19], and 12 months for 4 studies [20, 28, 29, 31].

Two studies asked the caregiver about hours spent on caregiving in a typical day [18, 24], and 4 studies asked the caregiver about hours spent on caregiving in the last week before the interview [16, 17, 19, 31].

Nine studies used the opportunity cost approach to value informal care time [14, 15, 19, 23, 28–30, 32, 33]. Among them, 4 studies valued informal care time as leisure time with the disposable income of the caregiver [29, 30, 32, 33]. Four studies valued informal care time as productivity loss with the GDP per capita, the median salary declared by the caregiver, and the hourly wage rate [15, 19, 23, 28]. One study valued informal care time according to the caregiver type [14]. The GDP per capita was used to value time spent by retired caregiver and the global average daily salary in Great Britain to value time spent by working caregiver.

Eight studies used the replacement cost approach to value informal care time [13, 18, 20, 24–27]. Two studies used the hourly wage rate for housekeeping [13] and for a nurse working at a public hospital [26]. Two studies used the gross wage for a domestic cleaner [20] and the monthly salary [27]. Three studies valued informal care time according to the different tasks performed by the caregiver [18, 24, 25]. One study used the mean hourly wage rate of 4 at home workers as nurse, housekeeping, bookkeeping, and outdoor maintenance, to value informal care time [25]. One study used the hourly wage of home health aides to value time spent for ADL and the hourly wage of a homemaker to value the time spent for IADL [24]. Another one valued the time spent on care by the hourly wage of a home health and in home labour services and the time spent on supervision with the live-in employees working 55 hours a week [18].

Three studies used the replacement cost and the opportunity cost approaches to value informal care time [21, 22, 31]. Two studies used the replacement cost approach as the mean hourly wage rate of 4 at home workers as nurse, housekeeping, bookkeeping, and outdoor maintenance, to value informal care time, to value time spent by caregiver considered as family and friends and the opportunity cost approach as the mean hourly salary of other visitors than family and friends (e.g., clergy, teachers) [21, 22]. One study used the mean hourly wage rate for formal care declared by the caregiver to value the time spent on caregiving for a patient that used formal and informal care and used the mean hourly wage rate of the caregiver to value time spent for a patient that use only informal care [31].

Two studies used the opportunity cost approach as the average hourly salary of the caregiver to value time spent on caregiving by working caregivers and the contingent valuation or revealed preference analysis to value leisure time lost because of informal care by the retired caregivers [16, 17].

### 3.2. Alzheimer Disease

Among studies focused on AD, 12 [13–15, 18, 20–27] gave informal care time and costs for patients at home (Table 2). Among studies that included only one caregiver, informal care time ranged from 11.59 h to 47.60 h per week, and annually informal costs ranged from US$1,364 to US$10,752 [14, 15, 26, 27]. Informal care time ranged from 39.20 h to 80.00 h per week among studies that included several caregivers (i.e., >1) [13, 20–22, 25], and the annual informal costs associated ranged from US$10,700 to US$34,517. Among studies that did not precise the number of caregiver included, informal care time varied from 35.72 h to 139.30 h per week, and annual informal costs ranged from US$7,188 to US$34,163 [18, 23, 24]. Globally at home, the number of informal care hours was multiplied by 2.5 between mild (mean = 23.26 h/week) and severe stages (mean = 58.45 h/week) and the associated annual informal costs were multiplied by 4, from US$5,664 to US$20,029 between mild and severe stages. Informal costs accounted for 2.80% to 84.50% of total costs.

Five studies estimated informal care time and costs for institutionalized patients [21–23, 25, 27] (Table 3). Among the studies that included several caregivers, informal care time ranged from 8.90 h to 17.25 h per week and annually informal costs ranged from US$2,485 to US$5,542 [21, 22, 25]. In the study that included only one caregiver, the amount of informal care time was 3.02 h per week and the associated informal costs were US$416 annually [27]. In the study of Hux et al., where the number of caregiver was not defined, the amount of informal care time was 6.30 h per week and the associated informal costs were US$1,985 [23]. Globally in institution, the number of informal care hours was multiplied by 2.20 between mild (mean = 4.33 h/week) and severe stages (mean = 9.49 h/week) and the associated annual informal costs were multiplied by 2.10, from US$2,334 to US$4,911 annually. Informal costs accounted for 2.80% to 14.60% of total costs.

Five studies estimated informal care time and costs for patients without distinction between home and institution living [16, 17, 19, 23, 27] (Table 4). In the study that included only one caregiver, the amount of informal care time was 13.29 h per week and the associated informal costs were US$1,831 annually [27]. Informal care time ranged from 21.90 h per week to 66.50 h per week, and associated informal costs varied from US$4,428 to US$11,251 annually in the four studies that did not specify the number of caregivers [16, 17, 19, 23]. Globally, the number of informal care hours was multiplied by 0.70 between mild (mean = 18.53 h/week) and severe stages (mean = 27.19 h/week), and the associated annual informal costs were multiplied by 0.6, from US$4,483 to US$7,321 annually. Informal care costs accounted for 9.43% to 52.28% of total costs.

### 3.3. Parkinson Disease

Among studies focused on PD, 4 estimated informal care time and costs for patients at home [29–32] (Table 2). Among these, one study included only one caregiver, informal care time was 22.00 h per week, and associated informal costs were US$5,386 annually [31]. The other three studies did not specify the number of caregivers involved, and informal care time varied from 10.00 h to 21.20 h per week, with associated costs ranging from US$1,563 to US$3,832 annually [29, 30, 32]. According to studies that estimated precisely informal burden by severity level, the number of informal care hours and costs was 3 times higher in mild stage (hours = 10.00/week; costs = US$1.132) than in severe stage (hours = 30.80/week; costs = US$3,484) [29]. Globally, for patients at home, informal care costs account for 16.00% to 79.50%.

One study estimated informal care time and costs without defining the place of living [28] (Table 4). The number of informal time was 33.90 h per week, and informal costs accounted for US$19,413 annually. Informal care costs accounted for 43.40% of total costs.

### 3.4. Vascular Dementia

Informal care time, without distinction of place of living, varied from 32.82 h per week for mild stage to 25.27 h per week for severe stage [33] (Table 4). Associated informal costs varied from US$5,782 to US$7,508, respectively. Globally for all confounded severity level, informal care time was 39.20 h per week and informal costs were to US$8,969 annually. Informal care costs accounted for 20.80% of total cost.

### 3.5. Comparison of Informal Care Time and Costs between Dementias

#### 3.5.1. For Patients at Home

**Figure 2:**
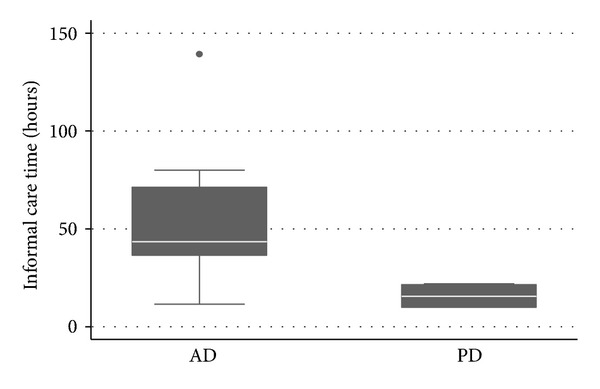
Time spent by caregiver (hours/per week) in AD and PD.

For patients at home, informal care time varied from 11.59 h per week to 80.00 h per week for AD and varied from 10.00 h per week to 22.00 h per week for PD (Figure 2). Mean informal care time was 55.73 ± 33.34 h per/week for AD and 15.80 ± 6.70 h per week for PD (*P* = 0.0076).

**Figure 3:**
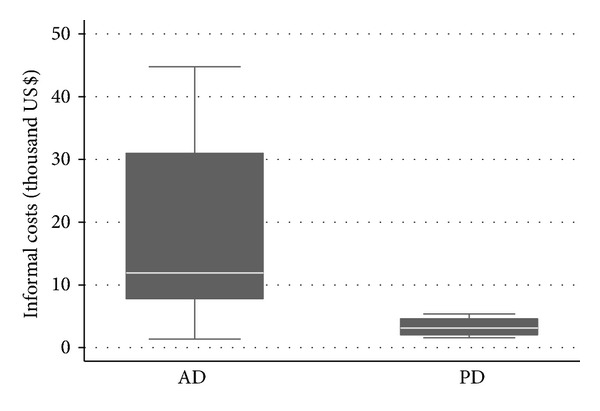
Informal costs (US$) in AD and PD.

For patients at home, informal costs varied from US$1,364 to US$44,736 in AD patients and varied from US$1,563 to US$5,386 annually in PD patients (Figure 3). Annual mean informal costs were US$17,492 ± 14,211 for AD and US$3,284 ± 1,680 for PD patients (*P* = 0.0393).

#### 3.5.2. Without Distinction of Place of Living

As only one study for PD and one for VD were available, no comparison between informal care time and costs was allowed [26, 31]. However, mean informal care time was 29.19 h/per week in AD, 33.90 h per week in PD and 39.20 h per week in VD. The associated costs were US$6,265, US$19,413, and US$8,969, respectively.

## 4. Discussion

We observed results about informal care time and costs only for AD and PD. Globally, informal care time and costs represent a greater burden in AD than in PD (55.73 h/week versus 15.80 h/week, resp., and US$17,492 versus US$3,284 resp.). In this paper, we were unable to compare informal care time and costs between all different major dementias because of the lack of data in VD, FTD, and DLB.

Informal care time and costs varied widely. Commenting on these quantitative results is problematic because significantly different approaches have been adopted to estimate informal costs. Informal caregiving time can be different depending on the number of caregiver involved and according to activities type included in the studies. In this paper, ten studies included several activities type as ADL, IADL, and supervision while three included only time spent on ADL/IADL and underestimate informal care hours. The difficulty lies also in the measurement of “joint production” which is a concept introduced by Juster and Stafford and which consists in combining informal care with nonmarket activities at the same time (household activities e.g., take advantage of shopping for the patient and for itself at the same time) [43]. On the other hand, it seems to be harder to combine informal care with paid job because employee must be in his/her workplace [59]. To facilitate this measure, when filling in the questionnaire, respondents have to take into account joint production, that is, the possibility to record simultaneous activities [45].

Another problem that is specific to the measurement of informal care is the separation between normal housework that somebody does anyway and “additional housework” that is due to the care demands of the care recipients [45].

In addition, all studies considered only the number of hours of informal care, and they omitted an important dimension which is the timing of care throughout the day and the fluctuations associated. Indeed, the fluctuations increase at specific moments like mealtimes and bedtime. This consideration introduces the notion of “time-bound” which is interested in distinguishing the tasks shiftable over the time—like household and organisation—and the tasks nonshiftable by nature—like personal care, eating, and taking medications. The nonshiftable activities might involve an additional source of opportunity costs because it must be provided at specific moments or times of the day. This account shows the importance of confronting the number of care hours with the type of informal care provided [59].

To value informal care time, if the opportunity cost approach is used, modifications of informal caregiving activities natures will not be taken into account. These changes can be considered if the replacement cost approach is used. A different hourly wage rate can be used according to activities performed by the caregiver. For example, time spent by caregiver on ADL tasks can be valued with the wage of home health aides and time spent on IADL tasks can be valued with the wage of a homemaker [60]. Moreover, opportunity cost approach underestimates women, elderly and minorities times who suffer from discrimination in the labour market; in these cases informal care time will be valued with leisure time which is a percentage of work time (usually between 25 and 33%) [61]. In fact, both methods present limitations. The cost opportunity means giving different values for similar activities depending on who does the work, and the cost replacement means the existence of a perfect replacement specialist on the market. Even if the opportunity cost and the replacement cost approaches are rather insensitive to the heterogeneity and dynamics of informal care, they are the more commonly used because of their simplicity of implementation. In other methods as WB, CM, or CVM, the well-being of the informal caregiver has to be assessed and this information is complicated, long, and expensive to collect in studies [55].

Even if the same approach was used, the hourly wage rate used to value informal time varies widely according to studies. In this paper, the hourly wage of informal caregiver varied from $4.10 to $19.18 [14, 15, 19, 23, 28–30, 32, 33] if the opportunity cost approach was used and $2.6 to $12.12 [13, 18, 20, 22, 24–27] if the replacement cost was used.

The different dementias described here have different causes. They affect different parts of the brain, which causes a different proportion of functional and cognitive impairment. This explains the different need for informal care in different dementias and thus different informal costs between AD and PD.

No papers were available about informal care time and costs for PD or VD patients in institution. Nevertheless, time spent on informal caregiving for AD patients was higher at home than in institution. If the place of living is not specified, time spent on informal caregiving and associated costs will be overestimated if only at home patients are included and underestimated if only institutionalized patients are taken into account.

We hypothesized that informal caregiver spent 48 weeks per year and 7 days per week on informal caregiving. We need this information to compare different unit costs and time spent per week on informal caregiving to show results on informal care costs and time in different age-related dementias. This is a strong assumption, but no article was available on the time spent per year on informal caregiving tasks. We took 48 weeks per year because we assume that caregiver could work and have only one month of holidays, so they would work 11 months per year [62]. During these “holidays” informal caregivers can rely on respite structures, other informal or formal caregivers. We took seven days a week because, even if employees work 5 or 6 days a week, we considered that informal caregiver cannot let his/her related alone during the weekend. We are aware that informal care time is different between severity stages, so for the mild stage maybe we overestimate informal care time and for the moderate and severe stages we underestimate informal caregiving time [63].

In the study of van den Berg and Spauwen with the diary method, informal caregivers report that in 9.11 h per day of informal care they spent 3.42 h per day for ADL tasks [45]. Based on these results, we can estimate that more than 1/3 of caregiver's time is devoted to tasks like ADL. Being by nature nonshiftable, these activities are more stringent for caregivers with employment. If the formal care covers these kinds of activities, which represent third of the hours of informal care, this would reduce the burden on informal caregivers and could allow the decrease of early institutionalization [4, 5]. This issue could lead on the one hand to an increase in employment opportunity in personal assistance area and on the other hand to an increase in labour market participation of informal caregivers [59].

Most of the studies reviewed here took a follow-up period that did not exceed one year. The one-year follow-up period is short to assess chronic diseases costs that have an impact more heavily on society as far as the physical or psychological disabilities progress. However, data collection over a long period is difficult so the use of models may compensate this difficulty.

There is a lack of specific criteria to diagnose each dementia described here. Indeed, specific diagnosis criteria are well defined for AD and PD [8, 9, 37, 38], and this is not necessarily the case for the other dementias [64]. This would explain the difficulty to build studies on specific dementia that are difficult to diagnose with diagnostic criteria that we currently have.

In this paper, we focused on objective burden (in relation to the time spent on informal caregiving). It is interesting to note that there is a distinction between objective and subjective burdens [65]. The last one refers to different impacts: physical, psychological, emotional and so on. Mental illnesses, like dementia, are particularly impacted by subjective burden. The study of Hastrup et al. has been shown, using Caregiver Strain Index in which mental illnesses are associated with a higher burden compared with somatic diseases. The subjective burden is impacted by the objective burden. Indeed, the number of hours per week spent on informal caregiving had a statistically significant impact on the subjective burden of caregiver. The more the number of informal care hours increases, the more the subjective burden is important. Beyond 50 hours per week, subjective burden is greatly increasing [65]. In this paper of the literature, we did not focus on this type of information. However, informal care provides to caregivers positives aspects [66].

For all these reasons, the results seem difficult to generalize and then limit the scope for international comparison. Economic results are difficult to compare on account of monetary issues, such as fluctuating exchange rates and different purchasing powers of currencies. Domestic characteristics also affect resources consumption and unit costs, including differences in clinical practice and health care system framework.

In our paper, only transversal studies were included. This analysis of informal care time and costs could be completed by wide analysis including all kind of studies (i.e., longitudinal, cost-effectiveness, cost utility).

Despite its limitations, this paper has the merit of showing that informal care time and costs are insufficiently considered although they play an important role in economic burden to society and families.

## 5. Conclusion

Although PD informal care time and costs are lower than AD informal care time and costs, both dementias represent a significant economic burden to society. The preoccupation of economists on the burden of the disease joined the policies and caregivers preoccupations. This collective awareness can be a basis for decision-making. An effort should be made by the scientific community to estimate the economic burden of informal care in different dementias. This would provide information to allow a better decision-making about public health priorities in dementias.

## Figures and Tables

**Table 1:** Studies characteristics.

	Studies	Countries	Year	Dementia criteria	Severity criteria	Care setting	Age (mean year)	Sample size	Number of caregivers	Informal care measure instrument	Activity type	Number of patients using informal care	Followup (months)
	Rigaud et al. [13]	France	2003	NINCDS-ADRDA	MMSE	Home	80.50	48	>1	Questionnaire	ADL, IADL, supervision	NS	6
	Souêtre et al. [14]	UK	1999	NINCDS-ADRDA	MMSE	Home	79.13	128	1	Questionnaire	NS	NS	NS
	Souêtre et al. [15]	France	1995	NINCDS-ADRDA	MMSE	Home	65.95	51	1	Questionnaires	NS	NS	3
	Jönsson et al. [16]	Europe	2006	NS	MMSE	Home and institution	75.90	204	NS	RUD	ADL, IADL, supervision	NS	NS
	Mesterton et al. [17]	Sweden	2010	NS	MMSE	Home and institution	79.50	233	NS	RUD	ADL, IADL, Supervision	NS	12
	Cavallo and Fattore [18]	Italy	1997	NS	APA	Home	73.60	423	NS	Questionnaire	ADL, IADL, supervision	NS	NS
	Coduras et al. [19]	Spain	2009	NINCDS-ADRDA	CDR	Home and institution	76.70	560	NS	RUD	ADL, IADL, supervision	NS	NS
AD	Bastida et al. [20]	Spain	2006	NS	CDR	Home	75.50	237	>1	Questionnaire	ADL	NS	12
	Max et al. [21]	USA	1995	NS	MMSE	Home and institution	78.00	NS	>1	Calendar	ADL, IADL, supervision	NS	12
	Rice et al. [22]	USA	1993	NS	MMSE	Home and institution	NS	187	>1	Calendar	ADL, IADL, supervision	NS	12
	Hux et al. [23]	Canada	1998	NS	MMSE	Home and institution	NS	948	NS	Questionnaire	ADL, IADL, supervision	NS	6
	Leon and Neumann [24]	USA	1999	NS	MMSE	Home	79.4	150	NS	Questionnaire	ADL, IADL	NS	12
	Schnaider Beeri et al. [25]	Israel	2002	NS	MMSE	Home and institution	[76.40; 81.50]	121	>1	Questionnaire	ADL, IADL, supervision	NS	6
	Zencir et al. [26]	Turkey	2005	NS	MMSE	Home	70.50	42	1	Daily Time sheet	ADL	NS	0.5
	Allegri et al. [27]	Argentina	2006	NINCDS-ADRDA	MMSE	Home and institution	74.50	100	1	Question-naire	NS	NS	3

	Findley et al. [28]	UK	2010	NS	Hoehn and Yarh	Home and institution	71.70	302	NS	Questionnaire	NS	NS	5
	Keränen et al. [29]	Finland	2003	NS	Hoehn and Yarh	Home	66.50	258	NS	Questionnaire	NS	36%	12
PD	Winter et al. [30]	Czech Republic	2003	NS	NS	Home	63.60	100	NS	Questionnaire	NS	NS	6
	Whetten-Goldstein et al. [31]	USA	1997	NS	NS	Home	71.77	168	1	Questionnaire		34%	12
	Winter et al. [32]	Russie	2009	UKPDS	Hoehn and Yarh	Home	68.90	100	NS	Questionnaire	NS	42%	6

VD	Wimo and Winblad [33]	Sweden	2003	NS	NS	Home and institution	NS	NS	NS	RUD	ADL, IADL, supervision	NS	12

>1: several caregivers.

AD: Alzheimer disease; PD; Parkinson disease.

RUD: Resource Utilization in Dementia; ADL: activities of daily living; IADL: instrumental activities of daily living.

NS: not specified.

**Table 2:** Informal time and costs in at home patients.

Disease	Study				Mild			Moderate		Moderate to severe				Severe			Total
		Unit costs	Informal care time	Informal costs	Percent of TC	Informal care time	Informal costs	Percent of TC	Informal care time	Informal costs	Percent of TC	Informal care time	Informal costs	Percent of TC	Informal care time	Informal costs	Percent of TC
	Rigaud et al. [13]	7.84	7.08	2,666	33.50	28.42	10,696	53.00				120.00	45,225	67.00	39.20	14,743	51.00
	Souêtre et al. [14]	4.71	34.05	7,698	74.20	44.54	10,070	62.70				64.11	14,493	68.00	47.60	10,752	67.50
	Souêtre et al. [15]	1.75				26.60	2,108	41.00				31.50	2,768	33.00	29.05	2,438	38.80
	Cavallo and Fattore [18]	6.70													139.30	44,736	84.50
	Bastida et al. [20]	7.24	56.00	13,676	71.40	77.00	24,973	76.20				84.00	42,817	98.60	80.00	27,751	76.80
AD	Max et al. [21]	10.05				66.15	31,937	NS				75.43	36,389	NS	71.50	34,517	NS
	Rice et al. [22]	9.99				66.60	31,937	80.70				75.88	36,389	69.10	71.25	34,163	73.30
	Hux et al. [23]	4.20	22.05	7,248	NS	43.23	14,220	NS				46.75	15,372	NS	37.34	12,280	NS
	Leon and Neumann [24]	6.25	31.33	9,384	62.90	40.80	12,228	63.40				49.60	14,868	57.50	38.42	11,520	NS
	Schnaider Beeri et al. [25]	3.30													67.50	10,700	60.30
	Zencir et al. [26]	2.60	1.22	145	8.20	12.47	1,468	38.20				21.07	2,480	50.30	11.59	1,364	39.00
	Allegri et al. [27]	2.87	24.40	3,361	NS	39.50	5,441	NS				43.80	6,033	NS	35.90	4,940	2.80

	Keränen et al. [29]	2.36	10.00	1,132	NS							30.80	3,484	NS	21.20	2,395	16.00
PD	Winter et al. [30]	3.25													10.00	1,563	23.70
	Whetten-Goldstein et al. [31]	5.10													22.00	5,386	79.50
	Winter et al. [32]	7.99													10.00	3,832	27.90

NS: Not specified; % of TC: percentage of total costs; unit costs: valuation of one hour of informal caregiving.

All costs are in 2012 USD; Informal care time: per week; Informal costs: per year; unit cost: unit cost of one hour of informal care.

AD: Alzheimer disease; PD: Parkinson disease.

**Table 3:** Informal time and costs in institutionalized patients.

Disease	Study			Mild			Moderate		Moderate to severe				Severe			Total
		Unit costs	Informal care time	Informal costs	Percent of TC	Informal care time	Informal costs	Percent of TC	Informal care time	Informal costs	Percent of TC	Informal care time	Informal costs	Percent of TC	Informal care time	Informal costs
	Max et al. [21]	12.82				4.70	2,901	NS				9.30	5,728	NS	9.00	5,542	NS
	Rice et al. [22]	12.97				4.66	2,901	6.00				9.20	5,728	11.00	8.90	5,542	11.60
AD	Hux et al. [23]	6.50				3.62	1,200	NS	4.52	1,488	NS	9.97	3,276	NS	6.30	1,985	NS
	Schnaider Beeri et al. [25]	3.00													17.25	2,485	14.60
	Allegri et al. [27]	2.87													3.02	416	2.80

NS: not specified; Percent of TC: percentage of total costs; unit costs: valuation of one hour of informal caregiving.

All costs are in 2012 USD; informal care time: per week; informal costs: per year; unit cost: unit cost of one hour of informal care.

AD: Alzheimer disease.

**Table 4:** Informal time and costs without distinction of place of leaving.

Disease	Study			Mild			Moderate		Moderate to severe				Severe			Total	
		Unit costs	Informal care time	Informal costs	Percent of TC	Informal care time	Informal costs	Percent of TC	Informal care time	Informal costs	Percent of TC	Informal care time	Informal costs	Percent of TC	Informal care time	Informal costs	Percent of TC
	Jönsson et al. [16]	4.57	13.92	2,694	30.80	26.57	5,954	44.90	44.98	9,611	29.40	49.80	11,085	20.50	30.24	6,635	26.70
	Mesterton et al. [17]	6.60	10.30	3,104	13.20	14.20	5,871	10.30				20.20	4,218	5.90	13.97	4,428	9.40
AD	Coduras et al. [19]	3.94													66.55	11,251	52.30
	Hux et al. [23]	6.83	22.10	7,248	76.70	26.40	8,652	53.90	24.08	7,896	30.70	15.12	4,956	13.50	21.90	7,182	32.60
	Allegri et al. [27]	2.87	13.50	1,860	35.20	14.88	2,050	30.90				11.50	1,584	14.10	13.29	1,831	23.70

PD	Findley et al. [28]	18.53				31.74	18,548	56.70	34.82	21,708	40.30				33.90	19,413	43.40

VD	Wimo and Winblad [33]	4.77	32.82	7,508	17.60	47.57	10,882	26.30				25.27	5,782	12.30	39.20	8,969	20.80

NS: not specified; Percent of TC: percentage of total costs; unit costs: valuation of one hour of informal caregiving.

All costs are in 2012 USD; informal care time: per week; informal costs: per year; unit cost: unit cost of one hour of informal care.

AD: Alzheimer disease; PD: Parkinson disease; VD: vascular dementia.

## References

[2] Langa KM, Chernew ME, Kabeto MU (2001). National estimates of the quantity and cost of informal caregiving for the elderly with dementia. *Journal of General Internal Medicine*.

[3] van den Berg B, Brouwer WBF, Koopmanschap MA (2004). Economic valuation of informal care: an overview of methods and applications. *The European Journal of Health Economics*.

[4] Verbeek H, Meyer G, Leino-Kilpi H (2012). A European study investigating patterns of transition from home care towards institutional dementia care: the protocol of a RightTimePlaceCare study. *BMC Public Health*.

[5] Hébert R, Dubois MF, Wolfson C, Chambers L, Cohen C (2001). Factors associated with long-term institutionalization of older people with dementia: data from the Canadian study of health and aging. *Journals of Gerontology A*.

[6] Alzheimer’s Disease International

[7] Mauskopf J, Mucha L (2011). A review of the methods used to estimate the cost of Alzheimer’s disease in the United States. *American Journal of Alzheimer’s Disease and other Dementias*.

[8] McKhann G, Drachman D, Folstein M (1984). Clinical diagnosis of Alzheimer’s disease: report of the NINCDS-ADRDA work group under the auspices of Department of Health and Human Services Task Force on Alzheimer’s disease. *Neurology*.

[9] Gibb

[10] Román GC, Tatemichi TK, Erkinjuntti T (1993). Vascular dementia: diagnostic criteria for research studies: report of the NINDS-AIREN International Workshop. *Neurology*.

[11] McKeith IG, Galasko D, Kosaka K (1996). Consensus guidelines for the clinical and pathologic diagnosis of dementia with Lewy bodies (DLB): report of the consortium on DLB international workshop. *Neurology*.

[12] The Lund and Manchester Groups (1994). Clinical and neuropathological criteria for frontotemporal dementia. *Journal of Neurology, Neurosurgery & Psychiatry*.

[13] Rigaud AS, Fagnani F, Bayle C, Latour F, Traykov L, Forette F (2003). Patients with Alzheimer’s disease living at home in France: costs and consequences of the disease. *Journal of Geriatric Psychiatry and Neurology*.

[14] Souêtre E, Thwaites RM, Yeardley HL (1999). Economic impact of Alzheimer’s disease in the United Kingdom: cost of care and disease severity for non-institutionalised patients with Alzheimer’s disease. *British Journal of Psychiatry*.

[15] Souêtre EJ, Qing W, Vigoureux I (1995). Economic analysis of Alzheimer’s disease in outpatients: impact of symptom severity. *International Psychogeriatrics*.

[16] Jönsson L, Jönhagen ME, Kilander L (2006). Determinants of costs of care for patients with Alzheimer’s disease. *International Journal of Geriatric Psychiatry*.

[17] Mesterton J, Wimo A, By Å, Langworth S, Winblad B, Jönsson L (2010). Cross sectional observational study on the societal costs of Alzheimer’s disease. *Current Alzheimer Research*.

[18] Cavallo MC, Fattore G (1997). The economic and social burden of Alzheimer disease on families in the Lombardy Region of Italy. *Alzheimer Disease and Associated Disorders*.

[19] Coduras A, Rabasa I, Frank A (2010). Prospective one-year cost-of-illness study in a cohort of patients with dementia of Alzheimer’s disease type in Spain: the ECO study. *Journal of Alzheimer’s Disease*.

[20] Bastida JL, Serrano-Aguilar P, Perestelo-Perez L, Oliva-Moreno J (2006). Social-economic costs and quality of life of Alzheimer disease in the Canary Islands, Spain. *Neurology*.

[21] Max W, Webber P, Fox P (1995). Alzheimer’s disease: the unpaid burden of caring. *Journal of Aging and Health*.

[22] Rice DP, Fox PJ, Max W (1993). The economic burden of Alzheimer’s disease care. *Health Affairs*.

[23] Hux M, O’Brien B, Iskedjian M, Goeree R, Gagnon M, Gauthier S (1998). Relation between severity of Alzheimer’s disease and costs of caring. *Canadian Medical Association Journal*.

[24] Leon J, Neumann PJ (1999). The cost of Alzheimer’s disease in managed care: a cross-sectional study. *The American Journal of Managed Care*.

[25] Schnaider Beeri M, Werner P, Adar Z, Davidson M, Noy S (2002). Economic cost of Alzheimer disease in Israel. *Alzheimer Disease and Associated Disorders*.

[26] Zencir M, Kuzu N, Beşer G, Ergin A, Çatak B, Şahiner T (2005). Cost of Alzheimer’s disease in a developing country setting. *International Journal of Geriatric Psychiatry*.

[27] Allegri RF, Butman J, Arizaga RL (2007). Economic impact of dementia in developing countries: an evaluation of costs of Alzheimer-type dementia in Argentina. *International Psychogeriatrics*.

[28] Findley L, Wood E, Lowin J, Roeder C, Bergman A, Schifflers M (2011). The economic burden of advanced Parkinson’s disease: an analysis of a UK patient dataset. *Journal of Medical Economics*.

[29] Keränen T, Kaakkola S, Sotaniemi K (2003). Economic burden and quality of life impairment increase with severity of PD. *Parkinsonism & Related Disorders*.

[30] Winter Y, Campenhausen S, Brozova H (2010). Costs of Parkinson’s disease in Eastern Europe: a Czech cohort study. *Parkinsonism & Related Disorders*.

[31] Whetten-Goldstein K, Sloan F, Kulas E, Cutson T, Schenkman M (1997). The burden of Parkinson’s disease on society, family, and the individual. *Journal of the American Geriatrics Society*.

[32] Winter Y, Campenhausen S, Popov G (2009). Costs of illness in a Russian cohort of patients with Parkinson’s disease. *PharmacoEconomics*.

[33] Wimo A, Winblad B (2003). Societal burden and economics of vascular dementia: preliminary results from a Swedish-population-based study. *International Psychogeriatrics*.

[34] Quentin W, Riedel-Heller SG, Luppa M, Rudolph A, König HH (2010). Cost-of-illness studies of dementia: a systematic review focusing on stage dependency of costs. *Acta Psychiatrica Scandinavica*.

[35] Mauskopf J, Racketa J, Sherrill E (2010). Alzheimer’s disease: the strength of association of costs with different measures of disease severity. *The Journal of Nutrition, Health & Aging*.

[36] Herman N, Tam DY, Balshaw R (2010). The relation between disease severity and cost of caring for patients with Alzheimer disease in Canada. *Canadian Journal of Psychiatry*.

[37] Crum RM, Anthony JC, Bassett SS, Folstein MF (1993). Population-based norms for the mini-mental state examination by age and educational level. *Journal of the American Medical Association*.

[38] Hoehn MM, Yahr MD (1967). Parkinsonism: onset, progression and mortality. *Neurology*.

[39] Small GW, McDonnell DD, Brooks RL, Papadopoulos G (2002). The impact of symptom severity on the cost of Alzheimer’s disease. *Journal of the American Geriatrics Society*.

[40] Zhu CW, Scarmeas N, Torgan R (2006). Longitudinal study of effects of patient characteristics on direct costs in Alzheimer disease. *Neurology*.

[41] Érsek K, Kovács T, Wimo A (2010). Costs of dementia in Hungary. *The Journal of Nutrition, Health & Aging*.

[42] O’Shea E, O’Reilly S (2000). The economic and social cost of dementia in Ireland. *International Journal of Geriatric Psychiatry*.

[43] Juster FT, Stafford FP (1991). The allocation of time: empirical findings, behavioral models, and problems of measurement. *Journal of Economic Literature*.

[44] Robinson JP, Juster FT, Stafford FP (1985). The validity and reliability of diaries versus alternative time use measures. *Time, Goods, and Well-Being*.

[45] van den Berg B, Spauwen P (2006). Measurement of informal care: an empirical study into the valid measurement of time spent on informal caregiving. *Health Economics*.

[46] Clipp EC, Moore MJ (1995). Caregiver time use: an outcome measure in clinical trial research on Alzheimer’s disease. *Clinical Pharmacology and Therapeutics*.

[47] Davis KL, Marin DB, Kane R (1997). The Caregiver Activity Survey (CAS): development and validation of a new measure for caregivers of persons with Alzheimer’s disease. *International Journal of Geriatric Psychiatry*.

[48] Wimo A, Jonsson L, Zbrozek A (2010). The Resource Utilization in Dementia (RUD) instrument is valid for assessing informal care time in community-living patients with dementia. *The Journal of Nutrition, Health & Aging*.

[49] O’Shea E (2000). The costs of caring for people with dementia and related cognitive impairment. *Report*.

[50] van den Berg B, Brouwer W, van Exel J, Koopmanschap M, van den Bos GAM, Rutten F (2006). Economic valuation of informal care: lessons from the application of the opportunity costs and proxy good methods. *Social Science & Medicine*.

[51] Harrow BS, Tennstedt SL, McKinlay JB (1995). How costly is it to care for disabled elders in a community setting?. *Gerontologist*.

[52] Posnett J, Jan S (1996). Indirect cost in economic evaluation: the opportunity cost of unpaid inputs. *Health Economics*.

[53] Koopmanschap MA, van Exel NJA, van den Berg B, Brouwer WBF (2008). An overview of methods and applications to value informal care in economic evaluations of healthcare. *PharmacoEconomics*.

[54] van den Berg B, Ferrer-I-Carbonell A (2007). Monetary valuation of informal care: the well-being valuation method. *Health Economics*.

[55] van den Berg B, Brouwer W, van Exel J, Koopmanschap M (2005). Economic valuation of informal care: the contingent valuation method applied to informal caregiving. *Health Economics*.

[56] van den Berg B, Al M, Brouwer W, van Exel J, Koopmanschap M (2005). Economic valuation of informal care: the conjoint measurement method applied to informal caregiving. *Social Science & Medicine*.

[57] Hicks JR (1939). The foundations of welfare economics. *The Economic Journal*.

[58] Zay N (1981). *Dictionnaire-Manuel de Gérontologie Sociale*.

[59] Hassink W, van den Berg B (2011). Time-bound opportunity costs of informal care: consequences for access to professional care, caregiver support, and labour supply estimates. *Social Science & Medicine*.

[60] Leon J, Cheng CK, Neumann PJ (1998). Alzheimer’s disease care: costs and potential savings. *Health Affairs*.

[61] Segel JE Cost-of-Illness studies-A primer.

[62] Ray R, Schmitt J (2007). No-vacation nation: a comparison of leave and holiday in OECD countries. *European Economic and Employment Policy Brief*.

[63] Bakker C, de Vugt ME, van Vliet D The use of formal and informal care in early onset dementia: results from the NeedYD Study.

[64] Hachinski V (2008). Shifts in thinking about dementia. *Journal of the American Medical Association*.

[65] Hastrup LH, van den Berg B, Gyrd-Hansen D (2011). Do informal caregivers in mental illness feel more burdened? A comparative study of mental versus somatic illnesses. *Scandinavian Journal of Public Health*.

[66] Szmukler G (1996). From family ‘burden’ to caregiving. *The Psychiatrist*.

